# Enhanced Photodynamic Therapy Efficacy through Solid Lipid Nanoparticle of Purpurin-18-N-Propylimide Methyl Ester for Cancer Treatment

**DOI:** 10.3390/ijms251910382

**Published:** 2024-09-26

**Authors:** Sooho Yeo, Huiqiang Wu, Il Yoon, Hye-Soo Kim, Young Kyu Song, Woo Kyoung Lee

**Affiliations:** 1 Yonsei Institute of Pharmaceutical Sciences, College of Pharmacy, Yonsei University, 85 Songdogwahak-ro, Yeonsu-gu, Incheon 21983, Republic of Korea; sooho32@yonsei.ac.kr (S.Y.); hskim6181@gmail.com (H.-S.K.); 2 Center for Nano Manufacturing and Department of Nanoscience and Engineering, Inje University, Gimhae 50834, Republic of Korea; wuhuiqiang7@163.com (H.W.); yoonil71@inje.ac.kr (I.Y.); 3 Research Center of Dr. i&B Co., Daejeon 34047, Republic of Korea

**Keywords:** photodynamic therapy, photosensitizers, purpurin-18-N-propylimide methyl ester, solid lipid nanoparticle

## Abstract

Photodynamic therapy (PDT) is an innovative cancer treatment that utilizes light. When light irradiates, purpurin-18-N-propylimide methyl ester (P18 N PI ME) generates reactive oxygen species that destroy cancer cells. The hydrophobic nature of P18 N PI ME presents challenges regarding its aggregation in the body, which can affect its effectiveness. This study aimed to enhance the bioavailability and effectiveness of cancer treatment by synthesizing P18 N PI ME and formulating P18 N PI ME-loaded solid lipid nanoparticles (SLNs). The efficacy of PDT was estimated using the 1,3-diphenylisobenzofuran (DPBF) assay and photocytotoxicity tests on the HeLa (human cervical carcinoma) and A549 (human lung carcinoma) cell lines. The P18 N PI ME-loaded SLNs demonstrated particle sizes in the range of 158.59 nm to 248.43 nm and zeta potentials in the range of –15.97 mV to –28.73 mV. These SLNs exhibited sustained release of P18 N PI ME. DPBF analysis revealed enhanced PDT effects with SLNs containing P18 N PI ME compared with standalone P18 N PI MEs. Photocytotoxicity assays indicated toxicity under light irradiation but no toxicity in the dark. Furthermore, the smallest-sized formulation exhibited the most effective photodynamic activity. These findings indicate the potential of P18 N PI ME-loaded SLNs as promising strategies for PDT in cancer therapy.

## 1. Introduction

Cancer, characterized by abnormal cell growth, poses a significant global health challenge. Normally, cells adhere to specific functions and growth patterns, but cancerous cells lose these regulatory mechanisms, proliferating uncontrollably and potentially spreading to adjacent tissues or distant locations [1]. Treatment options for cancer therapy encompass surgical intervention, radiation therapy, and medication employing anticancer agents such as chemotherapy [2]. Radiation therapy utilizes high-energy radiation to eradicate tumors or impede cell multiplication [3,4]. Chemotherapy, a prevalent anticancer approach, aims to eliminate cancer cells or curb their growth. This therapy was developed historically by screening natural or synthetic compounds with anticancer properties [5]. It operates through diverse mechanisms, targeting specific aspects or functions within cancer cells or impeding their division [5]. However, traditional chemotherapy drugs possess drawbacks like swift elimination, limited drug resistance due to non-specific distribution within the body, and the emergence of drug resistance.

Photodynamic therapy (PDT) is a treatment modality that eradicates tumors or aberrant cells by combining light with a specific drug known as a photosensitizer (PS) [6]. This method specifically targets cancer cells by administering PSs. The lipoproteins acting as transporters could combine with PSs and facilitate the delivery of PSs to the area of the tumor rather than normal cells [7,8,9]. PSs react upon receiving light of specific wavelengths, interacting with surrounding oxygen to produce singlet oxygen. The reactive oxygen species destroy surrounding abnormal cells, tumors, or cancer cells while safeguarding relatively normal cells from significant damage [6,7,8,9]. PDT is a non-invasive treatment capable of targeting cancer cells without resorting to surgery [6,10,11]. It exhibits minimal side effects and, at times, presents more efficacious results than conventional approaches like surgery or radiation therapy.

Purpurin-18-N-propylimide methyl ester (P18 N PI ME), one of the PSs that are highly hydrophobic, often encounters challenges related to its solubility and aggregation in the body [12]. Nanoparticle technology serves as a method to enhance the solubility of poorly soluble drugs [13]. Solid lipid nanoparticles (SLNs) are one of the nanoparticle technologies used in drug delivery systems. They encapsulate drugs within nanoparticles consisting of lipid components to enhance drug delivery and absorption [14]. SLNs are used in a variety of drug delivery systems, particularly in cancer treatment [14,15]. It is advantageous to improve anticancer effects by enhancing drug stability and delivery. SLNs can improve photostability, contributing to drug stability. The solid lipids in SLNs physically prevent external light from reaching the encapsulated drugs [16,17]. Regarding cancer treatment, lipid-based nanoparticle technologies are expected to enhance the pharmacological efficacy of P18 N PI MEs according to cellular uptake [16]. Previous studies on porphyrin or chlorin-class PS-encapsulated nanoparticles tended to enhance PDT effect [18,19,20].

The primary goal of this study was to enhance the bioavailability and the anticancer effects of PDT by synthesizing P18 N PI ME from chlorophyll and designing SLNs encapsulating P18 N PI ME. P18 N PI ME was synthesized from the chlorin class of PSs, which was expected to exhibit relatively long-wavelength absorption compared with porphyrin-class PSs such as hematoporphyrin derivatives and Photofrin [20,21]. Consequently, there is the possibility of effectively treating deeply placed tumors. We performed UV–vis spectroscopy for the detection of the absorption wavelength and ^1^H nuclear magnetic resonance (^1^H-NMR) spectroscopy for the structure of the synthesized P18 N PI ME. The pharmaceutical characteristics of the SLNs with P18 N PI ME were assessed by analyzing their particle size, zeta potential, and photostability. The pharmacological effects of PDT with SLNs with P18 N PI ME were determined by measuring ^1^O_2_ photogenesis using 1,3-diphenylisobenzofuran (DPBF) non-biological analysis and in vitro phototoxicity for human cervical carcinoma (HeLa) and human lung epithelial carcinoma (A549) using WST biological analysis due to the high cancer mortality rates [22]. Dark conditions for safety and lighting conditions utilizing LED lights capable of irradiating a broad spectrum of wavelengths were employed for these analyses.

## 2. Results and Discussion

### 2.1. Characterization of P18 N PI ME

#### 2.1.1. NMR Spectroscopy

**Figure 1:**
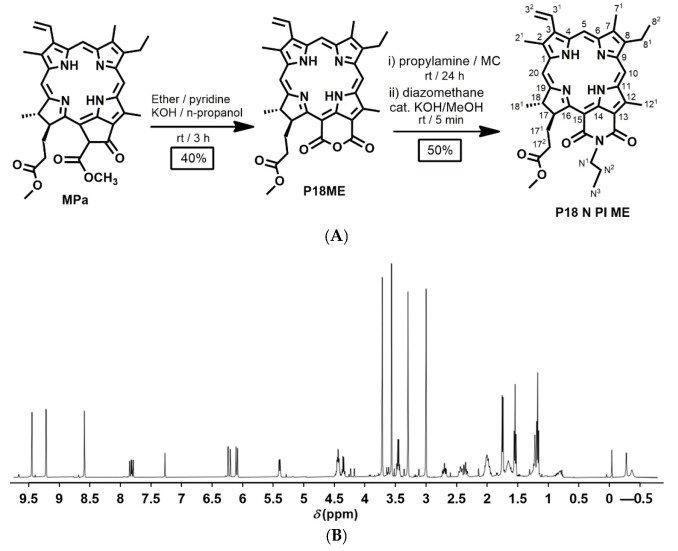
(**A**) Schematic representation of the synthesis of P18 N PI ME from P18ME; (**B**) ^1^H-NMR spectrum of P18 N PI ME (500 MHz, CDCl_3_, 25 °C, TMS).

The structure of P18 N PI ME was characterized by ^1^H-NMR spectroscopy. Figure 1 shows the ^1^H-NMR spectrum of P18 N PI ME. ^1^H-NMR (500 MHz, CDCl_3_, 25 °C, TMS): δ 9.40 (s, 1H, 10H), 9.17 (s, 1H, 5H), 8.55 (s, 1H, 20H), 7.79 (dd, *J* = 17.9, 11.6 Hz, 1H, 3^1^H), 6.22 (d, *J* = 18.0 Hz, 1H, 3^2^H), 6.09 (d, *J* = 11.5 Hz, 1H, 3^2^H), 5.39 (m, 1H, 17H), 4.44 (m, 2H, N^1^H), 4.36 (m, 1H, 18H), 3.72 (s, 3H, 12^1^H), 3.57 (s, 3H, OCH_3_), 3.47 (q, *J* = 7.7 Hz, 2H, 8^1^H), 3.30 (s, 3H, 2^1^H), 3.01 (s, 3H, 7^1^H), 2.71 (m, 1H, 17^2^H), 2.48–2.34 (m, 2H, 17^1^H), 2.08–1.95 (overlapped, m, 3H, N^2^H + 17^2^H), 1.78 (d, *J* = 7.4 Hz, 3H, 18^1^H), 1.57 (t, *J* = 7.7 Hz, 3H, 8^2^H), 1.20 (t, *J* = 7.4 Hz, 3H, N^3^H), and –0.23 and –0.33 (each br, 2H, NH).

#### 2.1.2. Development of Analytical Method for P18 N PI ME

##### The Absorption Spectrum and Specificity of P18 N PI ME

**Figure 2:**
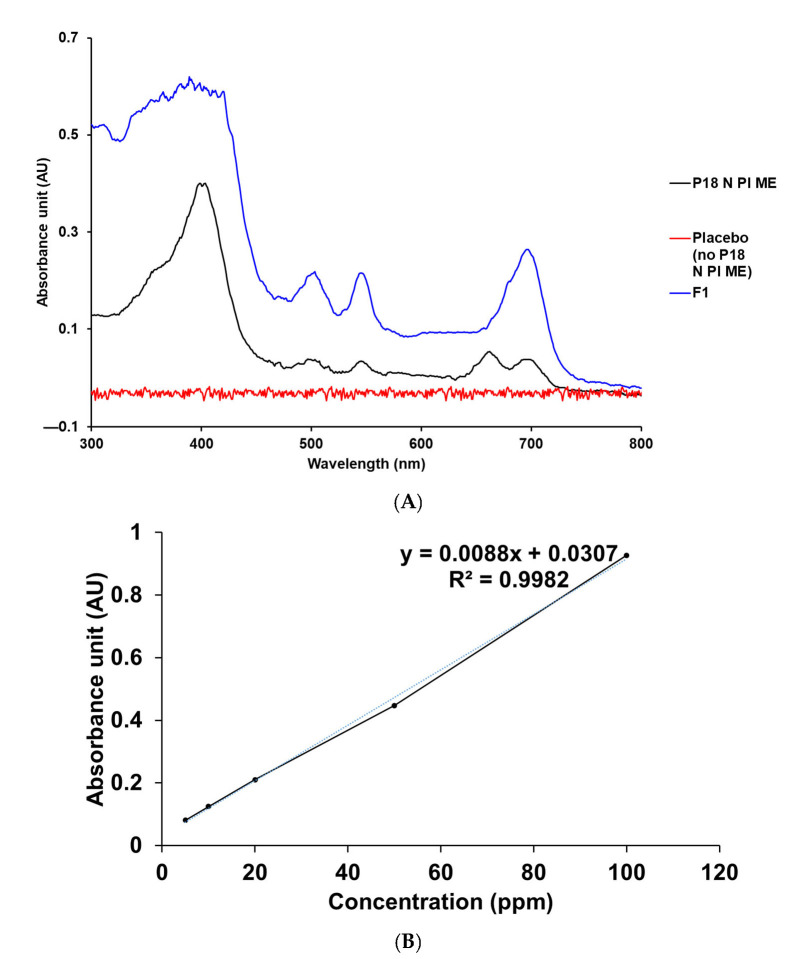
UV–vis spectrum and the calibration curve of P18 N PI ME. (**A**) Specificity data for P18 N PI ME, placebo (no P18 N PI ME), and P18 N PI ME-loaded SLNs in MeOH at 25 °C. (**B**) Linearity data for the standard solution of P18 N PI ME in MeOH.

To determine the specific absorption wavelength of P18 N PI ME, we evaluated its absorption spectrum and specificity using a UV–vis spectrophotometer [23,24,25]. Analysis of the UV–vis spectrum of P18 N PI ME revealed the maximum absorption wavelength at 707 nm, which was a relatively long wavelength compared with the MPa we previously studied at 664 nm (Figure 2) [25]. Examination of the UV–vis spectrum for the placebo SLN, which had the same composition as F1 but without P18 N PI ME, confirmed that the placebo did not interfere with the analyte. Consequently, P18 N PI ME was detected at a wavelength of 707 nm.

##### Linearity

To construct the calibration curve, we analyzed five standard stock solutions containing P18 N PI ME concentrations ranging from 5 to 100 ppm. The calibration curve, obtained through linear regression analysis, exhibited a notable correlation coefficient of 0.9982, as shown in Figure 2.

##### Precision

Precision is defined as the degree of agreement between measurements of different samples for a standard stock solution of the same concentration, as indicated by the relative standard deviation (RSD) of repeatability, calculated as the RSD (%) of absorption units. The precision results determined that the RSD (%) value of the recovery rate was 0.32%, as shown in Table 1. This result indicates the high precision of the proposed analytical method.

##### Accuracy

Accuracy, representing the degree of agreement between test outcomes and an established true or accepted reference value, was also quantified using the RSD derived from drug recovery. The obtained RSD (%) values for recovery, as shown in Table 2, were found to be 0.56%, 0.39%, and 0.18%. These results affirm the high accuracy achieved by the developed analytical method.

### 2.2. Characterization of P18 N PI ME-Loaded SLNs

#### 2.2.1. Nanoparticle Size, Polydispersity Index (PDI), and Zeta Potential

**Figure 3:**
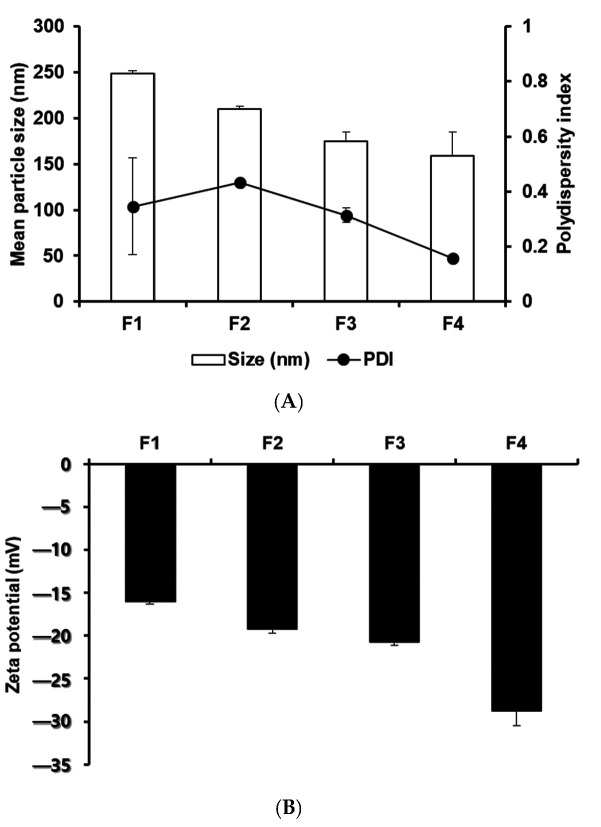
Particle characterization of P18 N PI ME-loaded SLNs manufactured using various components. (**A**) Particle size and polydispersity index (PDI) and (**B**) zeta potential. Results are presented as means ± standard deviation from three independent experiments (*n* = 3).

The particle size plays an important role in cancer treatment by influencing cellular uptake [26,27,28]. To reflect the stability of the particles, the zeta potential of the nanoparticles, representing the electrical surface potential, was measured [28,29]. Maintaining an optimal zeta potential in nanoparticles is crucial to prevent aggregation, ensuring stable storage and controlled drug release [30]. Therefore, balancing this potential is key to achieving a fine-tuned equilibrium between drug release and storage stability in the formulation [31]. As shown in Figure 3, the sizes of the F1, F2, F3, and F4 SLNs were 248.43 nm, 210.10 nm, 174.59 nm, and 158.59 nm, respectively. Additionally, the zeta potentials of those SLNs were −15.97 mV (F1), −19.20 mV (F2), −20.70 mV (F3), and −28.73 mV (F4). As a result of analyzing the particle size, PDI, and zeta potential, the F4 SLNs among formulations F1–F4 exhibited the smallest, most uniform, and most stable characteristics. Consequently, F4 seems to be a suitable choice for application in PDT based on cellular uptake. An analysis of the influence of different lipids indicated that formulations using longer-carbon-chain lipids exhibit a stronger affinity for P18 N PI ME, as the higher affinity between the drug and lipids facilitates the drug’s dissolution into the lipids during the formation of the O-phase of SLNs [30,32,33]. This, in turn, ensures that the drugs are molecularly dispersed within the lipids of the O-phase without interfering with the sonication energy used in SLN production [34]. The negative zeta potential was primarily attributed to the carboxyl group of the lipids, as the surfactant used was nonionic [35].

#### 2.2.2. Determination of Drug-Loading Capacity

**Figure 4:**
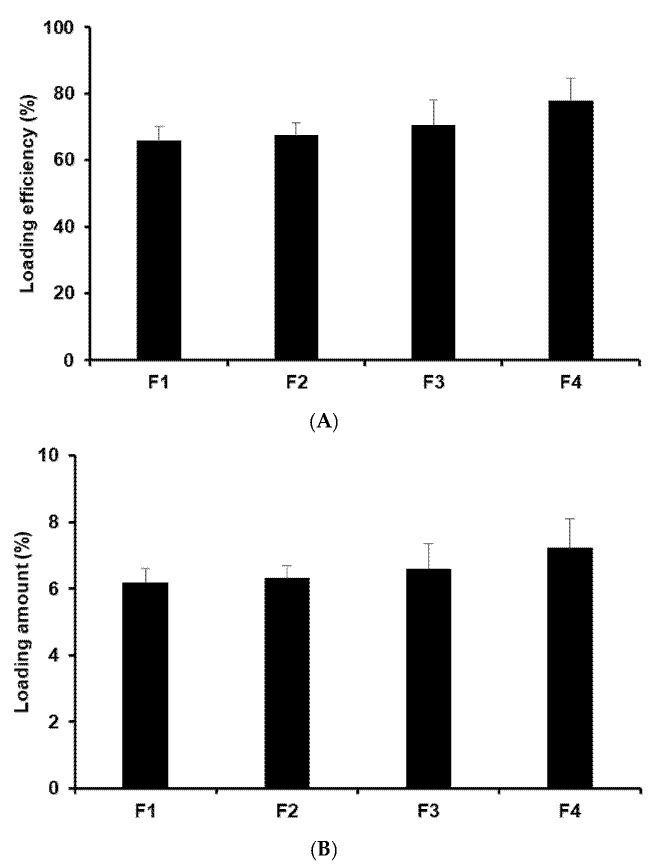
(**A**) Loading efficiency (LE) and (**B**) loading amount (LA) of P18 N PI ME in the formulations. Results are expressed as means ± standard deviation from three independent experiments (*n* = 3).

The drug-loading capacity is a crucial factor in drug delivery systems as it helps prevent side effects and degradation while enabling sustained drug release. In the case of the P18 N PI ME-loaded SLNs, the loading capacity (expressed as the LE and LA) ranged from 65.66% to 77.67% for the LE and from 6.16% to 7.21% for the LA, as shown in Figure 4. Among the formulations employing various lipids, there was a slight increase in the loading capacity of P18 N PI ME in the SLNs with longer-carbon-chain lipids. This implies that a strong affinity between P18 N PI ME and lipids enhances the solubility of P18 N PI ME within the lipid matrix, as corroborated by prior findings related to particle size and zeta potential. Regarding the effect of the surfactants, the formulation using Poloxamer 188 (PX 188) had higher amounts of loading efficiency (LE) and loading amount (LA) than that using Tween® 20 (TW 20). This suggests that the high hydrophilic–lipophilic balance (HLB) value of the surfactant affected the stable dispersion of the O-phase in the W-phase [36]. Furthermore, when considering the impact on the loading capacity, it was observed that lipids had a more pronounced effect compared with surfactants.

### 2.3. In Vitro P18 N PI ME Release Studies

**Figure 5:**
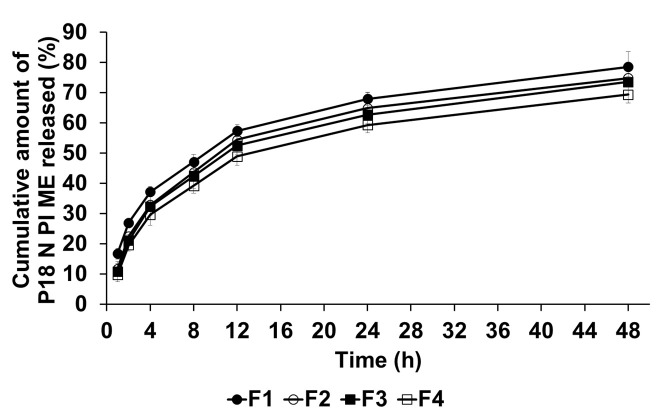
Cumulative release percentage profiles of P18 N PI ME from SLNs in the release medium, determined using the dialysis bag method. Results are presented as means ± standard error from three independent experiments (*n* = 3).

The release of P18 N PI ME from the SLNs was investigated through dialysis, and the results are shown in Figure 5. The release profiles of F1–F4 displayed a two-phase pattern, characterized by an initial burst release that lasted for more than 4 hours, followed by a sustained release extending up to 48 h. The order of release result after 48 h was F1 (78.57%) > F2 (74.79%) > F3 (73.53%) > F4 (69.40%). The initial burst release can be attributed to the adhesion of P18 N PI ME to the particle surface [37]. The delayed release suggests that lipids with a stronger affinity for P18 N PI ME restrained its release from the SLN core [38]. Concerning the impact of surfactants, enhancing the particle stability of the interfaces between the oil and water phases increased the drug-loading capacity of the SLNs, resulting in a delayed release of P18 N PI ME from the formulation [38].

### 2.4. Photostability Studies

**Figure 6:**
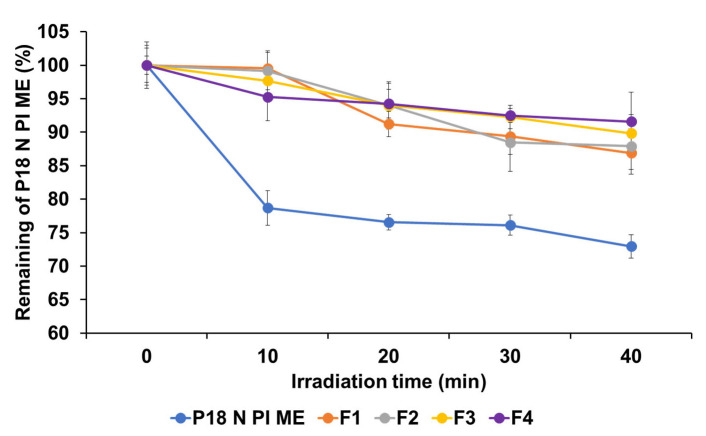
Photostability assessment of P18 N PI ME solution, using the percentage of non-degraded P18 N PI ME in both the solution and SLNs before and after LED irradiation. Results are expressed as means ± standard deviations of three independent experiments (*n* = 3).

In PDT, this is significant as the photostability of PSs is intricately linked to their pharmacological impact. An analysis of photostability revealed that all formulations contributed to the improved stability of P18 N PI ME when exposed to light, as shown in Figure 6. Following 40 min of photoirradiation, the order of photostability was found to be F4 > F3 > F2 > F1 > pure P18 N PI ME. The percentage of P18 N PI ME remaining in the P18 N PI ME solution was 72.97%, and that of the SLNs ranged from 86.90% to 91.59%. This suggests that the structural composition of the SLNs effectively shields the encapsulated drug from external light [39,40,41]. Additionally, the findings regarding photostability are aligned with the outcomes of the loading capacity experiment. This suggests that formulations exhibiting a higher encapsulation efficiency better shield drugs against the adverse effects of light [42]. In other words, high-stability particles can capture large amounts of drugs, effectively protecting them from light exposure.

### 2.5. ^1^O_2_ Photogeneration

**Figure 7:**
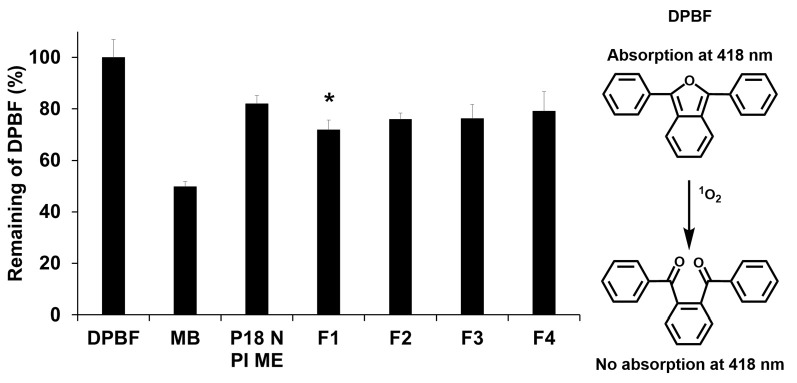
Reduction rate (%) of DPBF absorbance at 418 nm for P18 N PI ME with or without SLNs after light exposure (total light dose of 2 J/cm^2^; exposure time of 15 min). Statistical significance of the difference in DPBF degradation between P18 N PI ME and formulation is indicated by a single asterisk (*p* < 0.05) or double asterisks (*p* < 0.01). Results are shown as means ± standard deviation for triplicates (*n* = 3). NC: DPBF (1,3-diphenylisobenzofuran); PC: MB (methylene blue).

The PDT effect was evaluated through the DPBF assay, in which DPBF interacts with ^1^O_2_, leading to a reduction in the absorption band intensity of DPBF. To compare the pharmacological effects of PDT, we used methylene blue (MB) as a standard PS for 1O2. The DPBF results demonstrate that the SLNs of P18 N PI ME exhibited better 1O2 photogenesis than the P18 N PI ME solutions (Figure 7). The intensity values of the remaining DPBF for pure P18 N PI ME and the formulations were 82.00% and 71.88–79.19%, respectively. This means that ^1^O_2_ photogenesis for those test substances was 18.00% and 20.81–28.12%, respectively. This suggests that the utilization of SLNs prevents the aggregation of P18 N PI ME. The photodynamic efficiency of ^1^O_2_ in formulations F1–F4 followed the sequence F1 (28.12%) > F2 (23.96%) > F3 (23.73%) > F4 (20.81%). This sequence aligns with the observed release profile but contradicts the order of the zeta potential and photostability. Although it seems that the very stable formulations provide relatively low PDT efficacy, this might be due to the protective effect of the external light on the SLNs.

### 2.6. In Vitro Photocytotoxicity Studies

**Figure 8:**
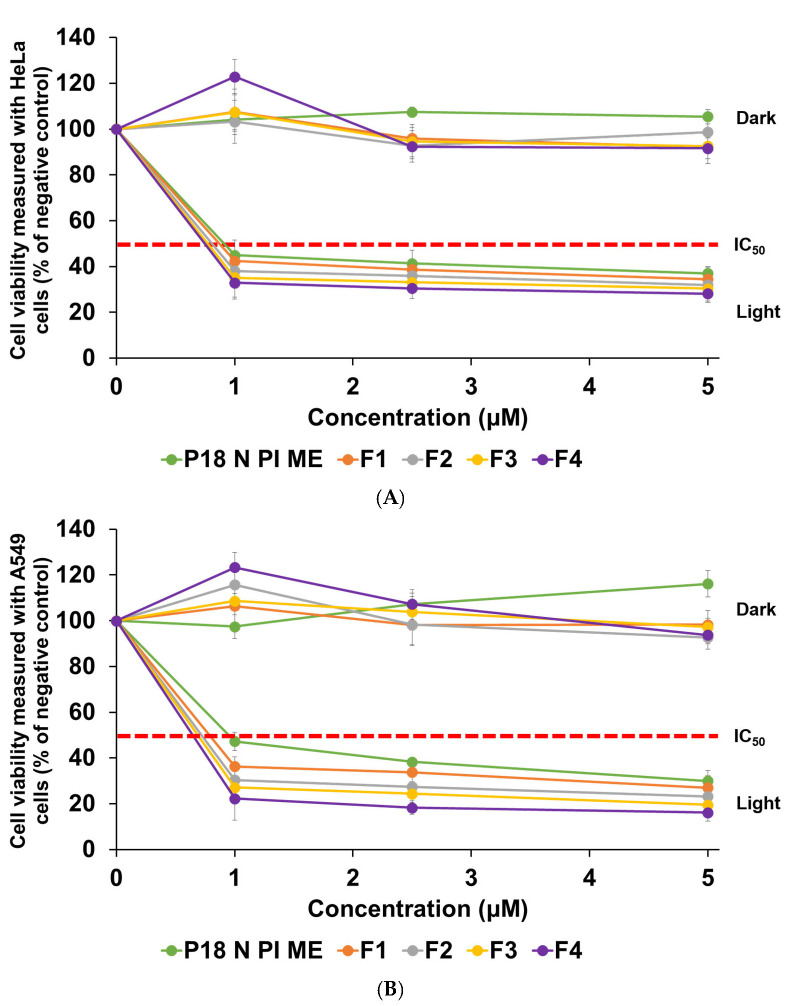
Cytotoxicity of P18 N PI ME solution and formulations F1, F2, F3, and F4 against (**A**) HeLa and (**B**) A549 cell lines. The WST assay was used for the estimation of viability. Results are exhibited as means ± standard deviation for triplicates (*n* = 3).

This study focused on examining the photocytotoxic effects of P18 N PI ME on human cervical carcinoma (HeLa) and human lung epithelial carcinoma (A549), evaluated as the PDT efficiency. The cell viability was assessed using the WST assay, as shown in Figure 8. We performed a cytotoxicity test to assess the safety (dark conditions) and anticancer effect (light conditions) of the formulations, as the mechanism of PDT involves the generation of ^1^O_2_ in the presence of light. The photocytotoxicity of P18 N PI ME showed anticancer effects according to the cell type and concentration of P18 N PI ME in all the test substances. We employed three varied concentrations (1, 2.5, and 5 µM) of each sample to determine the values for the 50% inhibitory concentration (IC_50_). The results of the IC_50_ are summarized in Table 3. Photocytotoxicity analysis was conducted to assess the impacts of varying lipids, namely, palmitic acid (PA) and glycerol monostearate (GMS), as well as different surfactants, including TW 20 and PX 188. In the absence of light, the viability of the HeLa cells and the A549 cells varied from 91.63% to 122.91% and from 92.71% to 123.28%, respectively. These results reveal that P18 N PI ME and SLN loading were not cytotoxic to the HeLa and A549 cell lines.

The evaluation of the two cancer cell lines’ viabilities following photoirradiation revealed that the anticancer effects of all the formulations depended on the concentration of P18 N PI ME, as shown in Figure 8. When comparing the IC_50_ values (expressed in μM), the order of PDT activity was found to be F4 (0.74 μM and 0.64 μM) > F3 (0.77 μM and 0.69 μM) > F2 (0.81 μM and 0.72 μM) > F1 (0.87 μM and 0.78 μM) > P18 N PI ME (0.91 μM and 0.95 μM) for the HeLa and A549 cell lines, respectively. In both cell lines, all the formulations demonstrated an enhancement in the anticancer effect compared with pure P18 N PI ME. The order of the PDT effect in all the formulations corresponded to the order of decreasing particle size. Among all the formulations, F4, which contained GMS (lipid) and PX 188 (surfactant), exhibited the most effective PDT outcome against both cell lines. This was attributed to its smallest particle size, promoting easy cellular uptake, subsequent drug release, and the generation of ^1^O_2_ [26,29,30,43]. Likely, the relatively high stability with a high zeta potential (–28.73 mV) of F4 facilitates minimum P18 N PI ME release in the blood. Typically, it is recognized that the particle size and loading capacity can impact cellular uptake and the expression of anticancer effects [43,44,45,46]. In this context, it appears that the influence of the particle size is more dominant than other factors when considering anticancer activity.

## 3. Materials and Methods

### 3.1. Materials

We obtained PA from Samchun Co. (Pyeongtaek, Republic of Korea). GMS was procured from Kanto Chemical Co., Ltd. (Tokyo, Japan). Chlorophyll-a paste was procured from Shandong Lanmo Biotech Co., Ltd. (Shanghai, China). PX 188 was obtained from BASF Co., Ltd. (Ludwigshafen, Germany). TW 20 was purchased from Dae Jung Co., Ltd. (Busan, Republic of Korea). Dulbecco’s modified Eagle’s medium (DMEM) was obtained from Welgene (Gyeongsan, Republic of Korea). Penicillin–streptomycin solution (100×) and fetal bovine serum (FBS) were obtained from BioWest (Nuaillé, France). Dimethyl sulfoxide (DMSO) for molecular biology, chloroform, phosphate-buffered saline (PBS), and MB were acquired from Sigma-Aldrich Co. (St. Louis, MO, USA). Methylene chloride (CH_2_Cl_2_ (MC)) was supplied by Duksan Co., Ltd. (Ansan-si, Gyeonggi-do, Republic of Korea). DPBF was supplied by TCI Chemicals (Tokyo, Japan). The cancer cell lines (HeLa and A549) were obtained from the Korean Cell Line Bank (Seoul, Republic of Korea), and the Quanti-MAX WST-8 assay kit was purchased from Biomax (Seoul, Republic of Korea). High-performance liquid chromatography (HPLC)-grade methanol (MeOH) was purchased from Honeywell (Seelze, Germany). All other chemicals used were of HPLC grade.

### 3.2. Synthesis of P18 N PI ME

Purpurin 18 methyl ester (P18ME) was synthesized via several steps following extracting chlorophyll-a paste based on our previously reported protocols [20,47]. P18ME (200 mg; 0.311 mmol) and propylamine (1.4 mL 20 eq) were dissolved in MC (10 mL) and stirred at 25 °C for 24 h. The color of the solution changed from pink to green. Diazomethane was added and stirred for about 5 min, and then the catalytic amount of KOH/MeOH was added. The color of the solution changed from green back to red. The reaction mixture was diluted with MC (100 mL) and washed with water and HCl, and the pink layer was dried with Na_2_SO_4_, concentrated, and purified by silica gel column chromatography (containing 0.1% Et_3_N) using acetone–MC = 2% as the eluent (yield: 50%).

### 3.3. Preparation of P18 N PI ME-Loaded SLNs

P18 N PI ME-loaded SLNs were prepared using a modified oil-in-water (O/W) emulsion method. Initially, 50 mg of either PA or GMS was dissolved at a temperature 10 °C above the melting point of the solid lipid, followed by the addition of 5 mg of P18 N PI ME to form the O-phase. A water phase was formed by dissolving 100 mg of TW 20 or PX 188 in water. The O-phase was then added to the W-phase to form the O/W emulsion, which was homogenized at 1000 rpm using a Polytron homogenizer (PT 3100; Kinematica Instruments, Luzerne, Switzerland). The resulting mixture was subjected to ultrasonic treatment using a probe sonicator (Scientz-IID, Ningbo, China) at 300 W for 15 min, with a 5 s pulse on and 5 s pulse off cycle, to achieve a nanoscale size. The compositions of the various P18 N PI ME-loaded SLNs are listed in Table 4.

### 3.4. Characterization of P18 N PI ME

#### 3.4.1. NMR Spectroscopy

The synthesized P18 N PI ME was characterized using a Varian spectrometer (500 MHz) for ^1^H-NMR at the Inje University Biohealth Products Research Center, South Korea. For the sample preparation, the compound was dissolved in CDCl_3_.

#### 3.4.2. Development of Analytical Method for P18 N PI ME

The content of P18 N PI ME was analyzed using a UV–vis spectrophotometer (S-3100, Scinco, Seoul, Republic of Korea). The absorbance spectrum of P18 N PI ME was measured in the wavelength range of 300 to 800 nm to determine the maximum absorbance. A standard stock solution was made by dissolving 2 mg of P18 N PI ME in 20 mL of MeOH. The interference effect (specific assessment) of the test results on the main component was confirmed by analyzing the standard stock solution, the test solution (containing the drug), and the placebo solution (drug-free sample).

##### Linearity

Five standard stock solutions ranging from 5 to 100 ppm were prepared via dilution in MeOH. In each formulation, calibration curves for P18 N PI ME were established correlating the concentration with the absorbance units.

##### Precision and Accuracy

The precision was determined by performing six repeated measurements, and the RSD was calculated. For accuracy, a recovery study was conducted by adding the reference value of the pre-analyzed samples at 0%, 20%, and 100% levels. The contents were measured in each UV–vis absorption spectrum.

### 3.5. Characterization of P18 N PI ME-Loaded SLNs

#### 3.5.1. Determination of Particle Characteristics

The characteristic analysis of the nanoparticles was performed using the dynamic light scattering (DLS) method at 25 °C with the Zetasizer Nano ZS (Malvern Instruments Ltd., Worcestershire, UK) equipment. Before measurement, all samples were pretreated by diluting them 10 times with distilled water (DW). The measurement results were calculated as the average ± standard deviation after measuring three test substances (*n* = 3).

#### 3.5.2. Determination of Drug-Loading Capacity

The LA and LE of P18 N PI ME in the SLNs were analyzed using a UV–vis spectrophotometer. The prepared nanoparticles were diluted 10 times with DW to prepare a 1 mL suspension. After centrifugation for 1 h at 4 °C at 1300 rpm, the P18 N PI ME content separated from the nanoparticles was analyzed. The measurement results were calculated as the average ± standard deviation after three measurements (*n* = 3). The LE and LA were calculated using Equations (1) and (2) as follows:

(1) $$LE\left(\%\right)=\frac{Amount\;of\;total\;drug\;content-Amount\;of\;free\;drug}{Amount\;of\;total\;drug\;content}\times 100$$

(2) $$LA\left(\%\right)=\frac{Amount\;of\;total\;drug\;content-Amount\;of\;free\;drug}{\left(Amount\;of\;total\;drug\;content-Amount\;of\;free\;drug\right)+Amount\;of\;lipid}\times 100$$

### 3.6. In Vitro Release Studies

A dialysis bag with a molecular weight cutoff of 10 kDa (Spectrum Laboratories, Inc., Compton, CA, USA) was soaked in primary distilled water for 12 h. The test substances were placed in the dialysis bags, and tongs were used to prevent any sample leakage. These sample-containing dialysis bags were inserted into vials, and 50 mL of PBS (pH 7.4) as the receptor solution was added. The vials containing the samples were shaken in a shaking incubator (JSSI-100T, JS Research Inc., Kongju, Republic of Korea) at a rate of 100 strokes per minute at 37 °C. At various measurement times (1, 2, 4, 8, 12, 24, and 48 h), 1 mL of the receptor solution was taken and filtered through a 0.45 µm syringe filter. The quantification of the measurements was performed by adding standard stock solutions and the corresponding samples with a UV–vis spectrophotometer.

### 3.7. Photostability Studies

The photostability of P18 N PI ME in the SLNs was conducted following a modified procedure detailed in the study by Jana et al. [44]. In a 20 mL solution containing 0.1% MeOH, pure drugs or formulations were prepared to achieve a concentration of 4.0 μg/mL. The manufactured samples were exposed to LED light (2 J/cm^2^), and the samples were collected at specified measurement intervals (0, 10, 20, 30, and 40 min). Vortexing was performed by adding 1 mL of hexane to dissolve the lipids. Subsequently, the layer containing the drug in 0.1% MeOH was separated, and the solution was filtered using a 0.22 μm syringe filter. The quantification of P18 N PI ME was carried out using a UV–vis spectrophotometer as described in Section 2.4.

### 3.8. ^1^O_2_ Photogeneration

Under light-shielding conditions, a 1 μM sample of each sample containing 50 μM of DPBF was added to DMSO. A negative control (NC) and a positive control (PC) were prepared with 50 μM of DPBF and 50 μM of DPBF with 1 μM of MB, respectively. Each sample was positioned within a 48-well plate and covered with aluminum foil for protection. The plate was irradiated with an LED at 2 J/cm^2^ for 15 min. A microplate reader (Synergy HTX, BioTek, Winooski, VT, USA) measured the absorbance at 418 nm.

### 3.9. In Vitro Photocytotoxicity Studies

The effectiveness of the nanoparticles in PDT was evaluated for two types of cancer cells: HeLa (from human cervical carcinoma) and A549 (from human lung carcinoma). The cancer cells were cultured in a 48-well plate at a concentration of 2 × 10^4^ cells/well in an environment of 5% CO_2_ and 37 ± 0.5 °C. The nanoparticles were added at various concentrations (1, 2.5, and 5 μM) and cultured for 24 h. After culturing, they were removed by washing with PBS, and 200 µL/well of the culture medium was added. To evaluate the PDT efficacy, LEDs were used to irradiate (2 J/cm^2^) the samples with light for 15 min at a height of 20 cm [47]. After culturing for 24 h, 10% WST-8 reagent was added at 100 µL/well, and the WST reaction was performed for 1 h. The absorbance was measured at 450 nm to evaluate the survival rate of the cells. The measurement results were calculated as the average ± standard deviation after three measurements (*n* = 3). The cell viability was calculated using the following Equation (3):

(3) $$Viability\left(\%\right)=\frac{{Mean\;OD}_{treated}}{{Mean\;OD}_{control}}\times 100$$

## 4. Conclusions

This study aimed to synthesize P18 N PI ME as a promising cancer treatment for PDT and designed P18 N PI ME-loaded SLNs to improve its light stability and pharmacological effects. The drug release analysis showed a two-phase pattern: an initial burst release lasting over 4 hours, followed by a continuous release extending up to 48 h. All P18 N PI ME-loaded SLNs exhibited significantly improved photostability and ^1^O_2_ photogeneration compared with free P18 N PI ME. The photoirradiation assessment involving two tumor cell lines (HeLa and A549) indicated that the P18 N PI ME-loaded SLNs were non-toxic in the absence of light but showed cytotoxicity when exposed to light. The formulations demonstrated improved anticancer effects compared with free P18 N PI ME. The PDT efficacy of the formulations was found to be influenced by the particle size. Among the different formulations, F4 demonstrated the most potent PDT effect on both cell lines, primarily due to its smaller particle size, facilitating retention at the tumor site by cellular uptake and efficient cellular uptake, subsequent drug release, and the generation of ^1^O_2_. Consequently, the findings from this study indicate that P18 N PI ME-loaded SLNs hold significant promise as anticancer agents for PDT.

## Figures and Tables

**Table 1:** Precision data were obtained with the developed analytical method for P18 N PI ME.

No	Recovery (%)
1	100.95
2	101.43
3	100.95
4	101.43
5	100.95
6	100.48
Average	101.03
SD	0.33
RSD	0.32

**Table 2:** Accuracy data were obtained with the developed analytical method for P18 N PI ME.

Drug (ppm)	No.	Recovery (%)	Average (%)	SD (%)	RSD (%)
5	1	103.70	103.29	0.58	0.56
	2	103.70			
	3	102.47			
20	1	101.43	100.95	0.39	0.39
	2	100.95			
	3	100.48			
100	1	99.57	99.75	0.18	0.18
	2	99.68			
	3	100.00			

**Table 3:** IC_50_ (μM) values, particle sizes, and loading efficiency (LE) values for P18 N PI ME solution and formulations F1, F2, F3, and F4 against HeLa and A549 cells.

Test Substance	HeLa (μM)	A549 (μM)	Particle Size (nm)	LE (%)
P18 N PI ME	0.91	0.95	N/A	N/A
F1	0.87	0.78	248.43 ± 3.47	65.66 ± 4.48
F2	0.81	0.72	210.10 ± 2.73	67.31 ± 4.02
F3	0.77	0.69	174.59 ± 10.16	70.44 ± 7.69
F4	0.74	0.64	158.59 ± 26.29	77.67 ± 6.87

N/A, not applicable.

**Table 4:** Compositions of SLNs loaded with P18 N PI ME.

	Drug (mg)	Lipid (mg)		Surfactant (mg)	
	P18 N PI ME	PA	GMS	TW 20	PX 188
F1	5	50		100	
F2	5	50			100
F3	5		50	100	
F4	5		50		100

P18 N PI ME, purpurin-18-N-propylimide methyl ester; PA, palmitic acid; GMS, glycerol monostearate; TW 20, Tween^®^ 20; PX 188, Poloxamer 188.

## Data Availability

The original contributions presented in the study are included in the article, further inquiries can be directed to the corresponding author/s.

## References

[1] Warburg O. (1956). On the origin of cancer cells. Science.

[2] Dabo-Trubelja A., Gottumukkala V. (2023). Review of cancer therapies for the perioperative physician. Perioper. Med..

[3] Baskar R., Dai J., Wenlong N., Yeo R., Yeoh K.-W. (2014). Biological response of cancer cells to radiation treatment. Front. Mol. Biosci..

[4] Rominiyi O., Vanderlinden A., Clenton S.J., Bridgewater C., Al-Tamimi Y., Collis S.J. (2021). Tumour treating fields therapy for glioblastoma: Current advances and future directions. Br. J. Cancer.

[5] Abbas Z., Rehman S. (2018). An overview of cancer treatment modalities. Neoplasm.

[6] Dolmans D.E., Fukumura D., Jain R.K. (2003). Photodynamic therapy for cancer. Nat. Rev. Cancer.

[7] Kurokawa H., Ito H., Matsui H. (2021). Porphylipoprotein accumulation and porphylipoprotein photodynamic therapy effects involving cancer cell-specific cytotoxicity. Int. J. Mol. Sci..

[8] Correia J.H., Rodrigues J.A., Pimenta S., Dong T., Yang Z. (2021). Photodynamic therapy review: Principles, photosensitizers, applications, and future directions. Pharmaceutics.

[9] Kwiatkowski S., Knap B., Przystupski D., Saczko J., Kędzierska E., Knap-Czop K., Kotlińska J., Michel O., Kotowski K., Kulbacka J. (2018). Photodynamic therapy–mechanisms, photosensitizers and combinations. Biomed. Pharmacother..

[10] Niculescu A.-G., Grumezescu A.M. (2021). Photodynamic therapy—An up-to-date review. Appl. Sci..

[11] Skalerič E., Petelin M., Gašpirc B. (2023). Antimicrobial photodynamic therapy in treatment of aggressive periodontitis (stage III, grade C periodontitis): A comparison between photodynamic therapy and antibiotic therapy as an adjunct to non-surgical periodontal treatment. Photodiagnosis Photodyn. Ther..

[12] Jiang L., Cao S., Cheung P.P.-H., Zheng X., Leung C.W.T., Peng Q., Shuai Z., Tang B.Z., Yao S., Huang X. (2017). Real-time monitoring of hydrophobic aggregation reveals a critical role of cooperativity in hydrophobic effect. Nat. Commun..

[13] Kumar S., Dilbaghi N., Saharan R., Bhanjana G. (2012). Nanotechnology as emerging tool for enhancing solubility of poorly water-soluble drugs. Bionanoscience.

[14] Scioli Montoto S., Muraca G., Ruiz M.E. (2020). Solid lipid nanoparticles for drug delivery: Pharmacological and biopharmaceutical aspects. Front. Mol. Biosci..

[15] Liu M., Wang F., Pu C., Tang W., Sun Q. (2021). Nanoencapsulation of lutein within lipid-based delivery systems: Characterization and comparison of zein peptide stabilized nano-emulsion, solid lipid nanoparticle, and nano-structured lipid carrier. Food Chem..

[16] Ma Y., Zhuang Z., Xing L., Li J., Yang Z., Ji S., Hu R., Zhao Z., Huo Y., Tang B.Z. (2021). The AIE-active dual-cationic molecular engineering: Synergistic effect of dark toxicity and phototoxicity for anticancer therapy. Adv. Funct. Mater..

[17] Sharifi M., Cho W.C., Ansariesfahani A., Tarharoudi R., Malekisarvar H., Sari S., Bloukh S.H., Edis Z., Amin M., Gleghorn J.P. (2022). An updated review on EPR-based solid tumor targeting nanocarriers for cancer treatment. Cancers.

[18] Lin X., Jiang X., Xu Y., Liu R., Zhang N., Li R., Xiang H., Zhao C., Zhao Z., Zeng W. (2023). Perfluorocarbon-encapsulated porphyrin-lipid nanoparticles as a photoactive pyroptosis inducer for cancer therapy. J. Biomed. Nanotech..

[19] Lima A.M., Dal Pizzol C., Monteiro F.B., Creczynski-Pasa T.B., Andrade G.P., Ribeiro A.O., Perussi J.R. (2013). Hypericin encapsulated in solid lipid nanoparticles: Phototoxicity and photodynamic efficiency. J. Photochem. Photobiol. B Biol..

[20] Yeo S., Song H.H., Kim M.J., Hong S., Yoon I., Lee W.K. (2022). Synthesis and design of purpurin-18-loaded solid lipid nanoparticles for improved anticancer efficiency of photodynamic therapy. Pharmaceutics.

[21] Kou J., Dou D., Yang L. (2017). Porphyrin photosensitizers in photodynamic therapy and its applications. Oncotarget.

[22] Cao W., Qin K., Li F., Chen W. (2024). Comparative study of cancer profiles between 2020 and 2022 using global cancer statistics (GLOBOCAN). J. Natl. Cancer Cent..

[23] Pi C., Zhao W., Zeng M., Yuan J., Shen H., Li K., Su Z., Liu Z., Wen J., Song X. (2022). Anti-lung cancer effect of paclitaxel solid lipid nanoparticles delivery system with curcumin as co-loading partner in vitro and in vivo. Drug Deliv..

[24] Sofiqul I., Murugan V. (2022). Development of validation of a rapid and simple analytical separation method for anticancer alkylating agents using application of total error concept. J. Res. Pharm..

[25] Yeo S., Yoon I., Lee W.K. (2022). Design and characterisation of pH-responsive photosensitiser-loaded nano-transfersomes for enhanced photodynamic therapy. Pharmaceutics.

[26] Diaz-Diestra D., Gholipour H.M., Bazian M., Thapa B., Beltran-Huarac J. (2022). Photodynamic therapeutic effect of nanostructured metal sulfide photosensitizers on cancer treatment. Nanoscale Res. Lett..

[27] Xie J., Wang Y., Choi W., Jangili P., Ge Y., Xu Y., Kang J., Liu L., Zhang B., Xie Z. (2021). Overcoming barriers in photodynamic therapy harnessing nano-formulation strategies. Chem. Soc. Rev..

[28] Gundogdu E., Demir E.-S., Ekinci M., Ozgenc E., Ilem-Ozdemir D., Senyigit Z., Gonzalez-Alvarez I., Bermejo M. (2022). An innovative formulation based on nanostructured lipid carriers for imatinib delivery: Pre-formulation, cellular uptake and cytotoxicity studies. Nanomaterials.

[29] Nelemans L.C., Gurevich L. (2020). Drug delivery with polymeric nanocarriers—Cellular uptake mechanisms. Materials.

[30] Sultana S., Alzahrani N., Alzahrani R., Alshamrani W., Aloufi W., Ali A., Najib S., Siddiqui N.A. (2020). Stability issues and approaches to stabilised nanoparticles based drug delivery system. J. Drug Target..

[31] Le T.N.Q., Tran N.N., Escribà-Gelonch M., Serra C.A., Fisk I., McClements D.J., Hessel V. (2021). Microfluidic encapsulation for controlled release and its potential for nanofertilisers. Chem. Soc. Rev..

[32] Singh A.K., Italiya K.S., Narisepalli S., Chitkara D., Mittal A. (2021). Role of chain length and degree of unsaturation of fatty acids in the physicochemical and pharmacological behavior of drug–fatty acid conjugates in diabetes. J. Med. Chem..

[33] Arellano H., Nardello-Rataj V., Szunerits S., Boukherroub R., Fameau A.-L. (2023). Saturated long chain fatty acids as possible natural alternative antibacterial agents: Opportunities and challenges. Adv. Colloid Interface Sci..

[34] Gershanik T., Benita S. (2000). Self-dispersing lipid formulations for improving oral absorption of lipophilic drugs. Eur. J. Pharm. Biopharm..

[35] Bnyan R., Khan I., Ehtezazi T., Saleem I., Gordon S., O’Neill F., Roberts M. (2018). Surfactant effects on lipid-based vesicles properties. J. Pharm. Sci..

[36] Ishak K., Annuar M.S.M., Ahmad N. (2017). Optimization of water/oil/surfactant system for preparation of medium-chain-length poly-3-hydroxyalkanoates (mcl-PHA)-incorporated nanoparticles via nanoemulsion templating technique. Appl. Biochem. Biotechnol..

[37] Yoo J., Won Y.-Y. (2020). Phenomenology of the initial burst release of drugs from PLGA microparticles. ACS Biomater. Sci. Eng..

[38] Zoubari G., Staufenbiel S., Volz P., Alexiev U., Bodmeier R. (2017). Effect of drug solubility and lipid carrier on drug release from lipid nanoparticles for dermal delivery. Eur. J. Pharm. Biopharm..

[39] Kumari A., Guliani A., Shukla A.K., Kumar S., Acharya A. (2020). Green surfactant based synthesis of curcumin loaded poly lactic-co-glycolic acid nanoparticles with enhanced solubility, photo-stability and anti-biofilm activity. J. Drug Deliv. Sci. Technol..

[40] Gerasimovich E., Kriukova I., Shishkov V.V., Efremov Y.M., Timashev P.S., Karaulov A., Nabiev I., Sukhanova A. (2024). Interaction of Serum and Plasma Proteins with Polyelectrolyte Microparticles with Core/Shell and Shell-Only Structures. ACS Omega.

[41] Gupta T., Singh J., Kaur S., Sandhu S., Singh G., Kaur I.P. (2020). Enhancing bioavailability and stability of curcumin using solid lipid nanoparticles (CLEN): A covenant for its effectiveness. Front. Bioeng. Biotechnol..

[42] Ioele G., Grande F., De Luca M., Occhiuzzi M.A., Garofalo A., Ragno G. (2021). Photodegradation of anti-inflammatory drugs: Stability tests and lipid nanocarriers for their photoprotection. Molecules.

[43] Sarella P.N.K., Vegi S., Vendi V.K., Vipparthi A.K., Valluri S. (2024). A Promising Frontier in Nanotechnology-based Drug Delivery. Asian J. Pharm. Res..

[44] Jana B., Thomas A.P., Kim S., Lee I.S., Choi H., Jin S., Park S.A., Min S.K., Kim C., Ryu J.H. (2020). Self-Assembly of Mitochondria-Targeted Photosensitizer to Increase Photostability and Photodynamic Therapeutic Efficacy in Hypoxia. Chem.-Eur. J..

[45] Li N., Sun C., Jiang J., Wang A., Wang C., Shen Y., Huang B., An C., Cui B., Zhao X. (2021). Advances in controlled-release pesticide formulations with improved efficacy and targetability. J. Agric. Food Chem..

[46] Cheng R., Xu T., Wang C., Gan C. (2021). The stabilization and antioxidant performances of coenzyme Q10-loaded niosomes coated by PEG and chitosan. J. Mol. Liq..

[47] Lee T.H., Liu Y., Kim H.J., Lee S.H., Song H.H., Shim Y.K., Lee W.K., Yoon I. (2021). Mitochondrial targeting cationic purpurinimide–polyoxometalate supramolecular complexes for enhanced photodynamic therapy with reduced dark toxicity. Eur. J. Inorg. Chem..

